# Impact of Ohmic Heating and High Pressure Processing on Qualitative Attributes of Ohmic Treated Peach Cubes in Syrup

**DOI:** 10.3390/foods9081093

**Published:** 2020-08-11

**Authors:** Massimiliano Rinaldi, Paola Littardi, Maria Paciulli, Tommaso Ganino, Emanuela Cocconi, Davide Barbanti, Margherita Rodolfi, Antonio Aldini, Emma Chiavaro

**Affiliations:** 1 Department of Food and Drug, University of Parma, Parco Area delle Scienze 27/A, 43124 Parma, Italy; massimiliano.rinaldi@unipr.it (M.R.); paola.littardi@unipr.it (P.L.); tommaso.ganino@unipr.it (T.G.); davide.barbanti@unipr.it (D.B.); margherita.rodolfi@studenti.unipr.it (M.R.); emma.chiavaro@unipr.it (E.C.); 2 National Research Council, Institute of BioEconomy (IBE), via Madonna del Piano, 10-50019 Sesto Fiorentino (FI), Italy; 3 Experimental Station for the Food Preserving Industry (SSICA), Viale Tanara, 31/a, 43121 Parma, Italy; emanuela.cocconi@ssica.it; 4 John Bean Technology SpA, Via Mantova 63/A, 43123 Parma, Italy; antonio.aldini@jbtc.com

**Keywords:** colour, high pressure processing, microstructure, ohmic heating, peach cubes, texture

## Abstract

Stabilization of ohmic pretreated peach cubes (*ohm*) in syrup, representative of semifinished fruit products, was finalized by ohmic heating (OHM) and high pressure processing (HPP), proposed respectively as thermal and nonthermal pasteurization, in comparison to a conventional pasteurization treatment (DIM). The samples were then studied in terms of histological, physical (dimensional distribution, tenderometry, texture, viscosity of syrup and colour), chemical (total phenolic and ascorbic acid content), and sensorial (triangle test) properties. Severe modifications of the cell walls were observed in *ohm*-DIM and *ohm*-OHM samples, with swelling and electroporation, respectively. From chemical analyses, significant reduction of ascorbic acid and simultaneous increase of total phenolics content were observed for *ohm*-DIM and *ohm*-OHM, probably in relation to the cell wall damages. *ohm*-HPP showed the best preservation of the dimensional characteristics and hardness, followed by *ohm*-OHM and *ohm*-DIM. In addition, textural and colour parameters evidenced similar results, with *ohm*-HPP as the less different from *ohm*. Finally, the sensorial analysis confirmed *ohm*-HPP and *ohm*-OHM samples as the most similar to *ohm* as well as the most appreciated in terms of colour and consistency.

## 1. Introduction

An important growing segment in the food market is represented by processed fruits with high nutritional value (antioxidants, vitamins, and/or specific bioactive compounds) and good sensory properties (colour, taste, and texture). It is well known that conventional thermal treatments can negatively affect these characteristics [1]. In recent years, technical advancements in food production, by the use of innovative technologies, improved fruit processing, also meeting the growing consumer’s interest in healthy, safe and sustainable foods [2]. Among the innovative technologies, ohmic heating [1,3] and high hydrostatic pressure [4] have been extensively studied on fruit products.

During ohmic heating (OHM), feasible only for foods with the right electrical conductivity, the electrical energy running through the products is converted into heat, both in batch or in continuous systems [5]. OHM eliminates variations in temperature throughout the material by a process known as “volumetric heating”. This procedure gives more uniform product heating as compared to other methods especially for high viscous or particulate products such as fruit pieces [1]. OHM is reported to be very effective on enzyme and microorganism’s inactivation, as compared to conventional heating [6]. Enzymes are very sensitive to the increase of the electric field strength (V/cm). Moreover, higher voltage gradients enhance the nonthermal effects of OHM on microorganisms, by amplifying electroporation. The frequency of the electric current and the modification of the electrochemical properties of food material, during OHM, also play an important role in microbial reduction [6]. The most important consequence of OHM on the physical properties of vegetables is represented by electroporation that could alter cells and tissues, with consequent implications on texture [7]. The extent of this phenomenon depends on the characteristics of the food material and on the process conditions such as temperature, applied frequency and voltage gradient [8]. Authors that worked on peach cubes recommended ohmic treatments at frequencies above 100 kHz to reduce or eliminate electroporation damages [9]. Moreover, fresh materials are reported to respond better to the ohmic treatment than those previously subjected to other processes or pretreatments [10].

High pressure processing (HPP), based on the use of hydrostatic pressures ranged between 100 and 1000 MPa, is generally proposed as nonthermal alternative to the traditional pasteurization, being able to stabilize foods without affecting their quality [11]. Mild pressures (<300 MPa) are indeed already able to inactivate microorganisms, while the use of temperature is requested for spore’s inactivation. On the other hand, enzymes can be very resistant to pressure, thus refrigeration temperatures, low pH, and antibrowning agents are often used to increase the shelf life of the HPP treated products [12]. Moreover, when applied to fruit or vegetables, HPP has a mild or no impact on low-molecular-weight compounds, related with the sensorial and healthy properties. However, HPP is reported to have an impact on other quality attributes such as texture, colour and flavour, depending on both process conditions and type of plant tissue [11]. Although this novel technology is being increasingly investigated, the main targets of research on plant-based foods are purees, sauces [13,14] or juices [15]. Only few studies have been focused on whole fruits and vegetables, or pieces of them, treated by HPP [16,17,18].

Tripathi et al. [19] evidenced that the qualitative response of processed fresh-cut fruits represents a complex task due to the effect of several factors (processing conditions, treatment, storage temperature, packaging conditions, microbial and physiological spoilage) on visual appearance, firmness, and consumer acceptance. The study of innovative technologies, able to preserve food quality, considering also the efficiency and sustainability of the processes, is thus of great relevance. In this regard, both ohmic heating and high pressure processing are reported to be green technologies: De Marco et al. [20] reported that ohmic heating followed by aseptic packaging has to be considered the preferred method to produce semifinished apricots from the environmental point of view; similarly, Cacace et al. [21] from LCA analysis concluded that HPP has a lower environmental impact if compared to conventional thermal pasteurization.

Thus, the aim of this research paper was to evaluate the effects of ohmic heating and high pressure processing on the physicochemical and microstructural properties of semifinished peach cubes and compare them to the conventional thermal pasteurization.

## 2. Materials and Methods

### 2.1. Samples, Preparation, and Storage

Peach cubes (10 mm side), *Prunus persica* (L.) Batsch, were obtained from Aspis, Hellenic Juice Industry (Argos, Greece). The cubes were thermal pre-treated by ohmic heating (F_70_ = 360 min) (*ohm*), in a 16° Bx sucrose solution with a 50% solid/liquid ratio. This sample was chosen in order to replicate the process to which a semifinished fruit product is subjected. As pretreatment was chosen Ohmic heating in order to better retain the structural quality of the samples. Peach samples were then subjected to different pasteurization treatments: conventional pasteurization (*ohm*-DIM), ohmic heating (*ohm*-OHM), and high hydrostatic pressure (*ohm*-HPP) to complete the sample stabilization. Three trials were carried out for each treatment.

The DIM treatment section had the following characteristics: stainless steel 304 dimpled tube, internal diameter 55 mm, total length 12 m, the flow rate was set to 1800 L/h. The outer and inner surface of the tube presented dimples and protrusions, respectively, that work as gentle vortex generators, which resulted in better fluid/solid mixing near the wall and higher turbulence. As peach cubes presented a pH ~ 3.6, *Lactobacillus plantarum* was considered as target microorganism and considering at least 5 decimal reductions at the center of the cubes, a minimum lethality value of 300 min at 70 °C was defined for the carrier solution. The treatment, conducted on peaches in syrup, was equal to 100 s at 98 °C representing the temperature of the holding tube. After the thermal treatment, the samples were continuously cooled in the aseptic portion of the plant, for about 5 min to reach 25 °C; then they were aseptically packed in 10 L bags (PE/MET/PE, thickness 77 µm).

After preliminary experiment, the HPP treatment was conducted at 600 MPa for 3 min, as suggested by other authors [22]. These treatment conditions were considered economically viable and microbiologically comparable to pasteurization. The treatment was conducted in a 300 L high pressure plant (Avure Technologies Inc., Middletown, Ohio, USA, model AV-30) at the “HPP Italia” facility of Traversetolo (Italy). Samples, at room temperature, were packed in PET bottles (250 mL) and cold water (4 °C) was used as pressure medium (temperature increase due to compression of about 2–3 °C/100 MPa). Treated samples, at a final temperature of about 4/5 °C, were then stored at refrigerated temperature (4 °C).

For the OHM treatment an ohmic heater, working at a flow rate of 1700 L/h, was brought to 98 °C. The product, at a starting temperature of 25 °C, was then sent to the same holding section used for DIM tests (98 °C, 100 s), obtaining the same lethality effect reported above. The required electrical power was calculated by considering the electrical conductivity of liquid and peach cubes (0.225 ± 0.030 and 0.199 ± 0.021 Sm^−1^ at 25 °C, respectively); it was measured on homogenized sample by means of a digital conductivity meter and in accordance with reported values [23]. The final temperature of the *ohm*-OHM treated cubes was 25 °C.

In addition, cook value $\left(C_{T_{ref}}^{z}\right)$ at the thermal center of the *ohm*-DIM and *ohm*-OHM treated samples was obtained from the integration of the time/temperature profiles measured during the tests. The heat penetration curves have been acquired by using K-type (Ni/Al-Ni/Cr) thermocouples placed at the center of the samples.

$$C_{T_{ref}}^{z}=\int_{0}^{t}10^{\left(T-T_{ref}\right)/z}dt$$

where: *t* = time (min), *T_ref_* = reference temperature; set equal to 100 °C, *z* = temperature increase that induces a 10-fold increase of the reaction rate of the chemical reaction taken as reference; *z* was set at 33 °C, as previously reported [24].

### 2.2. Histological Analysis

Peach samples were fixed in FAA solution (formalin: acetic acid: 60% ethanol solution, 2:1:17 *v*/*v*) [25]. After 2 weeks, they were dehydrated with gradual alcohol concentrations and, finally, they were included in a methacrylate resin (Technovit 7100, Heraeus Kulzer & Co., Wehrheim, Germany). The resulting blocks were sectioned at 3 μm thickness (transversal cuts) with a semithin Leitz 1512 microtome (Leitz, Wetzlar, Germany). The sections were stained with Toluidine Blue (TBO) solution [23] for the evaluation of structure and then observed by means of an optical microscopy Leica DM 4000 (Leica Imaging Systems Ltd., Wetzlar, Germany) equipped with a digital camera Leica DMC2900 (Leica Imaging Systems Ltd., Wetzlar, Germany). Six samples per treatment were observed.

### 2.3. Physical and Qualitative Analyses

The total soluble solids of syrup (° Brix) were determined in triplicate at 25 °C by refractometer (Model 2WAJ, Optika, Ponteranica, Italy). pH was measured in triplicate by means of a pH meter (Model 3150, Jenway, UK).

A laboratory vibratory sieve shaker with mesh sizes of 6.0, 4.0, 2.0, and 1.4 mm (Giuliani Tecnologie srl, Torino, Italy) was used to determine the size of peach pieces. Sieving analysis was performed three times on 1 kg of samples per treatment. Data are reported as percentage of the total number of pieces for each dimensional class.

Texture of all treated samples (*ohm*, *ohm*-DIM, *ohm*-HPP and *ohm*-OHM) was measured by TPA double compression test using a TA.XT2i Texture Analyzer (35 mm diameter cylindrical aluminium probe, with a pretest, test, and post-test speed of 1 mms^−1^ up to the 40% of the original sample height). The textural parameters considered were: hardness (maximum peak force of the first compression cycle, N), cohesiveness (ratio of positive force area during the second compression to that during the first compression area, dimensionless), resilience (ratio between the upstroke energy of the first compression by the downstroke energy of the first compression, dimensionless), and springiness (ratio between the time duration of force input during the second compression over that during the first compression, dimensionless) [26]. Ten samples for each trial were analyzed.

Tenderometer values were also measured on 200 g of washed cubes by means of Martin Tenderometer (FMC Food Machinery Parma, Italy) in triplicate [27].

Colour determination was carried out using a Minolta Colorimeter (CM 2600d, Minolta Co., Osaka, Japan) equipped with a standard illuminant D65, that simulates the daily light. The assessments were carried out on two sides of eight peach cubes. The CIElab colour space was used to estimate the colour values (CIE 1978) [28]. L* (lightness, black = 0, white = 100), a* (redness > 0, greenness < 0), b* (yellowness, b* > 0, blue < 0) were quantified on each sample using a 10 degrees’ position of the standard observer. The total colour change (ΔE) was calculated compared with the control sample using the following equation:

$$\Delta E=\sqrt{{\Delta L}^{*2}+\Delta a^{*2}+\Delta b^{*2}}$$

Sixteen samples of each trial were analyzed.

Viscosity of syrup was determined in triplicate with a rotational rheometer ARES-TA^®^ (Advanced Rheometric Expansion System) and the Orchestrator TM software. The study was performed in triplicate at 25 °C and at shear rates between 10 and 200 s^−1^ with the geometry of a concentric cylinder (cup diameter = 34 mm; concentric cylinder diameter = 32 mm and length = 33 mm). As the syrup was assumed to behave as non-Newtonian fluids, shear stress (*σ*) vs. shear rate (*γ*) curves were fitted by means of power-law model:

$$\sigma =k\cdot \gamma^{n}$$

where *k* = consistency coefficient (Pa s*^n^*) and *n* = flow behavior index (dimensionless).

### 2.4. Total Phenolic Content (TPC) and Ascorbic Acid Content

The total phenolic content (TPC) of the samples was determined according to the Folin–Ciocalteu colorimetric assay [29]. Briefly, 1 mL of test sample was added to 70 mL of distilled water and 5 mL of Folin–Ciocalteu’s phenol reagent (Sigma-Aldrich, Buchs, Switzerland) and vigorously shaken. After 5 min, 10 mL of a saturated solution of sodium carbonate was added and the sample was brought to a final volume of 100 mL with distilled water. Absorbance was measured after 60 min at 720 nm using a UV-Vis spectrophotometer (Shimadzu, Kyoto, Japan). Total phenols were expressed as mg per kilogram of (+)-catechin equivalents. Before the colorimetric assay samples were homogenized and suitably diluted with water.

Ascorbic acid determination was carried out by means HPLC-DAD following the method of Trifiro et al. [30] with some modifications. Liquid chromatographic analysis was performed by a Waters HPLC system consisting of: Alliance 2695 Sep. Module, Alliance column heater, 2996 photodiode array detector. Chromatographic separation was performed by a 250 × 4.6 mm, 5 μm Kinetex XB-C18 column (Phenomenex, Torrance, CA, USA) kept at 30 °C. A isocratic elution with 1.0 mL/min flow was applied. Eluent was water/0.3% metaphosphoric acid. Data acquisition and chromatograms integration as well as management of chromatographic system were performed using Empower 2.0 software (Waters). Acquisition wavelength: 254 nm. Before HPLC analysis, samples were suitably diluted with a 6% aqueous solution of metaphosphoric acid, homogenized using Ultra-Turrax (T25 basic IKA^®^, IKA-Werke, Staufen, Germany) and filtered with paper filter (Chemifarm, Parma, Italy) and with syringe drive filter unit 0.45 μm (MillexHA, Millipore, Millerica, MA, USA). As calibration standards, solutions containing 0.5, 1, 2, 5, 10, 20, 50, 100 mg/kg of L-ascorbic acid (Sigma-Aldrich, Bush, Switzerland) in 6% metaphosphoric acid were prepared starting from a 1000 mg/kg mother solution.

### 2.5. Sensorial Analysis

A consumer discriminant test was conducted with 30 untrained subjects (18 males, 22 females: average age 27 ± 6). The participants were asked to refrain from eating, smoking, drinking, or chewing gums for 1 h prior to testing. A triangle forced choice procedure [31] was used to determine differences between peach cubes from different treatments. Participants were requested to determine which sample was the odd one. Each sample was identified by a 3-digit code and the order of sample presentation was randomized according to the standard method UNE-EN ISO 4120:2008 [32]. An open-ended comment was also requested to the panelists. These comments can be helpful in understanding consumer’s preferences on the different samples.

### 2.6. Statistical Analysis

Means and standard deviations were calculated with SPSS (v. 26.0, SPSS Inc., Chicago, IL, USA) and the same software was used to perform one-way analysis (ANOVA) with Tukey post-hoc test to evaluate the significant differences among treatments (*p* < 0.05).

## 3. Results and Discussion

### 3.1. Histological Analysis

**Figure 1:**
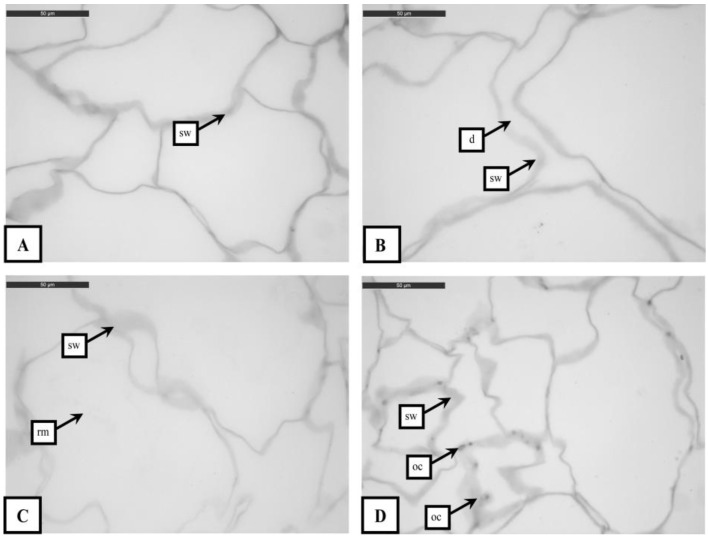
Transverse sections of peach samples subjected to different treatments and stained with Toluidine Blue: (**A**) *ohm*; (**B**) *ohm*-DIM; (**C**) *ohm*-HPP; (**D**) *ohm*-OHM. Legend: sw: swelling; d: cells detachment; rm: rupture of the cellular membrane; oc: opened channels in the cell wall. For *ohm* samples (**A**) thickening of the cell walls was visible due to the swelling phenomenon (sw). For *ohm*-DIM samples (**B**), cell swelling (sw) and detachment (d) were visible. On *ohm*-HPP samples (**C**), rupture of the membrane structure (spots inside of the cells—rm) and thickening of the cell wall due to swelling (sw) were observed. For *ohm*-OHM samples (**D**), cell walls showed thickened sections alternated with thin sections (oc) with consequent formation of channels; also in this case thickening of the cell walls (swelling) was observed.

Peach cubes were composed by only vascular bundles immersed in parenchymatic tissue. In sample *ohm*, cells appeared partially dehydrated due to the thermal treatment that peach cubes underwent before our experiment (Figure 1A). Some points of the cell walls appeared thickened. Thickening indicates swelling (sw) of the walls, probably due to the thermal degradation of the polymers and/or to changes in the ionic composition of the cell wall [33].

In *ohm*-DIM samples’ cells separation was visible (Figure 1B); cells detachment (d) was detectable as fissures in the parenchyma (Figure 1B). This phenomenon has been associated to breakage of chemical bonds between the pectic components of middle lamellae of adjacent cells and/or a hydrolysis of some other components of the cell wall (i.e., pectin, hemicelluloses, and cellulose) [34]. Cell walls did not show damages, but it was possible to observe the presence of thickened areas, probably due to swelling. The increase in the thickness/swelling was also observed in *ohm*-HPP-treated peach cubes (Figure 1C). According to other authors [35], cell wall alterations depend on an extensive strain exerted on the cell membrane and on the transmission of a considerable force onto these structures, caused by HPP treatment. HPP treatment did not substantially modify the microstructure compared to *ohm* (Figure 1A,C). The same results were observed on avocado [36] and carrots [37]. According to [38], HPP treated peaches cell morphology presents only minor changes of cellular shape and cell or tissue damages. On the contrary, Techakanon et al. [39] observed that cells changed from a cuboidal shape to a slightly spherical shape after HPP treatment. In our study, peach cubes did not show any change in shape, probably because the cubes were not from fresh but from semifinished product. Finally, a complete rupture of the cellular membrane occurred, visible for the presence of stains in the cells (rm), in agreement with Techakanon et al. [39] on peach.

Peach cubes treated with *ohm*-OHM showed altered cell walls (Figure 1D). The electropermeabilization mechanism seems to be the cause of these alterations [40]. A permeabilized cell shows opened channels, visible in Figure 1D as darker points (oc). This phenomenon allows diffusion of intracellular materials in the tissue, and in more severe treatments (50 °C) this alteration results in cell wall perforation, as previously demonstrated by Moreno et al. [40]. In our study, *ohm*-OHM peach cubes showed cell rupture due to retraction of cellular components (Figure 1D). Another effect of OHM treatment is the greatest thickness of the cell wall in comparison to other treatments. The intensity of the cell wall staining shows that this increase in size is mainly due to a modification of the composition probably due to the electropermeabilization effect.

### 3.2. Physico-Chemical Analyses

Total soluble solids content of the syrup resulted 9.57 ± 0.05, 9.50 ± 0.04, 9.53 ± 0.05 and 9.53 ± 0.03° Brix for *ohm*, *ohm*-DIM, *ohm*-HPP and *ohm*-OHM, respectively, with no significant differences between samples, as expected. Similarly, pH values of the homogenized whole product resulted in the range 3.5–3.6 with no significant differences among all treated samples. Peach cubes were immersed in an isotonic solution and mass exchanges were not possible.

### 3.3. Physical Analyses

**Figure 2:**
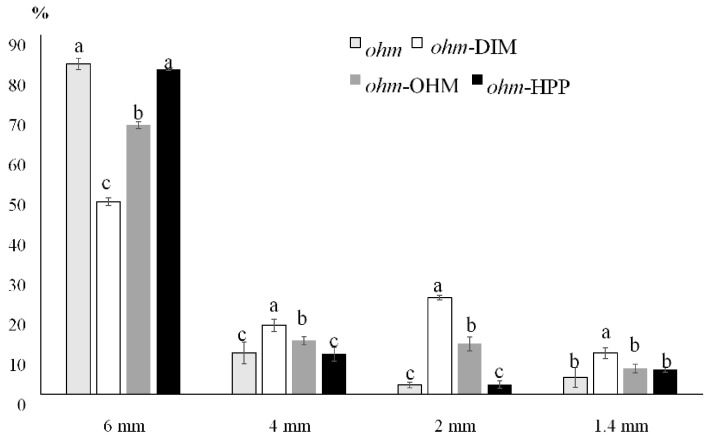
Percentage distribution of peach cubes among different sieves. Same letters within each sieve do not significantly differ (*n* = 3; *p* < 0.05).

Data obtained by sieving are reported in Figure 2. *ohm* and *ohm*-HPP samples presented the highest percentage of cubes with dimensions >6 mm. This result confirms that the loss of structure and the damage of the fruit tissues were considerably lower for *ohm*-HPP compared to other treatments [41]. In addition, *ohm*-OHM presented a high percentage of peach pieces with the biggest dimension, showing an advantage with respect to traditional heating technique. *ohm*-DIM samples presented the highest content of cubes retained by the smaller sieves (4.0, 2.0, and 1.4 mm) and the highest damages in the cubes’ structure, as a consequence.

The use of tenderometer, in addition to texture analysis, for the measurement of the mechanical properties of the peach cubes, was done because it is a very popular method in the fruit industry; we believe that readers from industry may be more familiar with the tenderometric units than with the texture results. Tenderometric units of *ohm* samples were 4.1 ± 0.3, *ohm*-HPP, *ohm*-OHM, and *ohm*-DIM gave instead values of 3.8 ± 0.2, 2.4 ± 0.2 and 1.6 ± 0.1, respectively. In accordance with sieving results, *ohm*-DIM samples were the most damaged while *ohm*-HPP samples were the least damaged ones, with *ohm*-OHM showing intermediate values. The obtained results may be due to the longer time needed by *ohm*-DIM cubes to achieve the required lethality value compared to *ohm*-OHM. Indeed, in the latter, a rapid and uniform treatment of liquid and solid phases with minimal damages to structure is generally reported [42], as confirmed by the calculated cook values (C_0_ 0.72 vs. 1.45 for *ohm*-OHM and *ohm*-DIM, respectively).

Textural parameters are reported in Table 1. As expected, hardness was higher for *ohm* and *ohm*-HPP samples, followed by *ohm*-OHM and *ohm*-DIM, in agreement with histological, tenderometric, and sieve analyses. Similarly, Zhang et al. [43] reported lower hardness for thermally treated yellow peach compared to the high pressure processed ones. *ohm*-OHM samples presented the typical damages of fruit tissue due to thermal treatments but in a lower extent compared to *ohm*-DIM, as also confirmed by the calculated cook values. Ohmic heating demonstrated advantage for the quality of peach cubes in syrup compared to the traditional thermal process. Regarding other textural parameters, *ohm*-DIM samples presented the lowest cohesiveness and resilience values while on the contrary *ohm*-HPP presented values similar to *ohm* ones in accordance with other studies [38]. For *ohm*-HPP treatment C_0_ value was 0 as product temperature never overcame 22 °C.

Interestingly, *ohm*-OHM treatment gave the highest springiness, being even higher than *ohm*: probably, the nonthermal effects of the ohmic treatment on cell walls through electroporation phenomenon could have generated a more deformable and elastic structure in this samples [8,40].

Table 2 showed significant differences between samples for all colorimetric parameters. In general, L* values significantly increased after all treatments if compared to *ohm* samples, in accordance with [44] on apricot dices. The increase of L* was higher for *ohm*-DIM and *ohm*-OHM, confirming the thermal degradation of pigments in these samples. On the contrary, *ohm*-HPP produced the lowest increase of this parameter (Table 2). The colorimetric parameter a* resulted slightly negative in *ohm*, showing a slight green component probably as a consequence of lye peeling [45]. *ohm*-DIM treatment caused a significant decrease in a* values while *ohm*-OHM a significant increase, as compared to *ohm*. Similar results were reported by Icier et al. [46] on pea puree with a tendency to redness with the increasing ohmic treatment time, possibly ascribable to nonenzymatic browning. Finally, *ohm*-HPP treatment did not affect a* coordinate, showing values not significantly different from *ohm*, in accordance with data reported by [38] on peach pieces. The parameter b*, following the same trend of L*, increased for both *ohm*-DIM and *ohm*-OHM, showing a more pale-yellow component on peach cubes’ surface after thermal treatments. On the contrary, *ohm*-HPP samples did not show significant differences compared to *ohm*. Finally, ΔE, expressing the total colour differences, was the highest in *ohm*-DIM and the lowest in *ohm*-HPP samples, as also previously reported [47].

**Figure 3:**
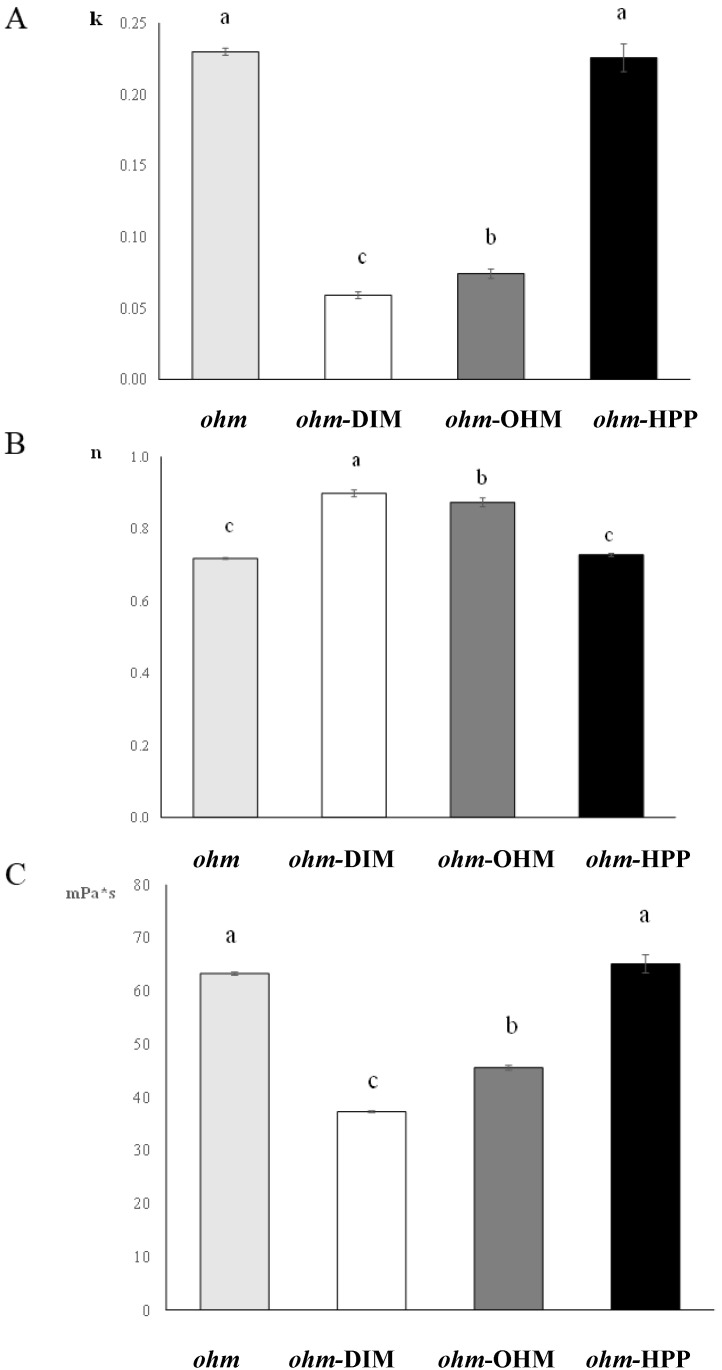
Consistency index (panel **A**), flow behavior index (panel **B**) and viscosity at 100 s^−1^ (panel **C**) of sugar syrup. ^a,b,c^ Same letters do not significantly differ (*n* = 3; *p* < 0.05).

For foods packaged in covering liquids and then processed, the flow behavior of the syrup influences the heat exchange mechanisms, thus the efficiency of the stabilizing treatments [48]. Less is known about the pressure/viscosity dependence of sugar solutions [49]. Figure 3 reports the rheological indexes of the sugar syrup as affected by the treatments.

The consistency coefficient (k) was significantly affected by *ohm*-DIM and *ohm*-OHM treatments while *ohm*-HPP samples did not show any significant difference with *ohm* (Figure 3A). In particular, *ohm*-OHM caused a significant decrease in k values, being even lower for *ohm*-DIM, with a consequent expected lower viscosity value.

Flow behavior index (n) is an opposite trend in comparison to k: thermal treatments (*ohm*-DIM and *ohm*-OHM) caused an increase of n values towards a value of 1, typical of Newtonian fluids, confirming damages to pectins in solution with loss of the shear thickening behavior (Figure 3B).

Viscosity of syrup measured at 100 s^−1^ (η 100) is reported in Figure 3C. *ohm*-DIM and *ohm*-OHM treatments caused a significant decrease of syrup viscosity probably due to β-eliminative degradation [50] and the very fast acid hydrolysis of water soluble pectins facilitated by the low pH [51]. On the contrary, viscosity was not significantly affected by *ohm*-HPP treatment confirming that this treatment caused a lower degradation of water soluble pectins in the covering liquid with no remarkable change in viscosity, in accordance with previous studies [52]. These results can be useful for either process design or new products formulation.

### 3.4. TPC and Ascorbic Acid Content

Ascorbic acid content was 450 ± 28 mg/kg_FW_ in *ohm* as, during the first treatment, producer added ascorbic acid as antibrowning agent. Reduction of ascorbic acid content resulted about 6%, 16%, and 22% for *ohm*-HPP, *ohm*-OHM, and *ohm*-DIM, respectively. Very low reduction of ascorbic acid in *ohm*-HPP is in agreement with [53] which observed similar reduction for minimally processed peaches subjected to 600 MPa of pressure. The higher ascorbic acid loss observed for *ohm*-DIM and in a lower extent for *ohm*-OHM may be ascribable to the thermal degradation, as already hypothesized for pigments.

Total phenolic content was 809 ± 56 mg/kg_FW_, measured as catechin equivalents. Differently from ascorbic acid, TPC showed a significant increase (about 50% more than *ohm*) after *ohm*-OHM and *ohm*-DIM treatments while no significant variations were observed for *ohm*-HPP (about 5%); probably, tissues’ damages in thermal treatments such as *ohm*-OHM and *ohm*-DIM could have caused the release of phenolic compounds in accordance with the histological analyses.

### 3.5. Sensorial Analysis

Consumer test evidenced that *ohm*-OHM sample was recognized by 19/30 panelists (significant level 0.1%) when compared to *ohm*-DIM sample, underling that *ohm*-OHM presented higher consistency and better colour. *ohm*-OHM was recognized by only 12/30 panelists when compared to *ohm* and *ohm*-HPP with no significant sensorial differences. Concluding, ohmic heating demonstrated a feasible technology for the thermal treatment of peach cubes, having also obtained good organoleptic response similar to the *ohm*-HPP ones.

## 4. Conclusions

High pressure and ohmic heating were applied on pretreated peach cubes in syrup to simulate an alternative to the detrimental heat treatment, conventionally applied as final processing step on some fruit products. High pressure was the treatment that better retained the overall quality of the peach cubes. Ohmic heating, although showing the limits of a heat treatment, resulted less invasive than the traditional thermal pasteurization. Moreover, ohmic heating led also to some interesting improvements, such as increased redness and total phenol content. Sensorial analysis confirmed that the ohmic-high pressure and ohmic-ohmic treated samples were more appreciated than the ohmic-thermal pasteurized ones. Concluding, high hydrostatic pressure and, secondly, ohmic heating could be considered feasible technologies for pretreated peach cubes in syrup, demonstrating advantage for the quality preservation of this semifinished product as compared to the traditional pasteurization process.

## Figures and Tables

**Table 1:** Textural parameters of peach cubes.

	Hardness (N)	Cohesiveness	Resilience	Springiness
*ohm*	3.23 ± 0.73 ^a^	0.164 ± 0.031 ^a^	5.58 ± 1.38 ^a^	54.9 ± 5.3 ^b^
*ohm*-DIM	0.95 ± 0.25 ^c^	0.135 ± 0.027 ^b^	2.92 ± 0.49 ^c^	56.7 ± 9.2 ^a,b^
*ohm*-OHM	2.48 ± 0.45 ^b^	0.138 ± 0.022 ^a,b^	4.31 ± 0.85 ^b^	63.6 ± 9.2 ^a^
*ohm*-HPP	3.43 ± 0.76 ^a^	0.152 ± 0.019 ^a,b^	5.11 ± 1.01 ^a,b^	54.2 ± 4.7 ^b^

^a,b,c^ Same letters within each column do not significantly differ (*n* = 10; *p* < 0.05).

**Table 2:** Colorimetric parameters of peach samples.

	L*	a*	b*	ΔE
*ohm*	72.5 ± 1.5 ^c^	−1.28 ± 0.27 ^b^	72.3 ± 1.3 ^b^	
*ohm*-DIM	80.4 ± 0.7 ^a^	−2.54 ± 0.21 ^c^	80.3 ± 2.5 ^a^	11.3 ± 0.31 ^a^
*ohm*-OHM	78.8 ± 0.6 ^a^	−0.67 ± 0.21 ^a^	78.0 ± 2.6 ^a^	8.6 ± 0.50 ^b^
*ohm*-HPP	76.3 ± 1.4 ^b^	−1.46 ± 0.40 ^b^	75.8 ± 1.6 ^b^	5.2 ± 0.95 ^c^

^a,b,c^ Same letters within each column do not significantly differ (*n* = 16; *p* < 0.05).
